# iSC.MEB: an R package for multi-sample spatial clustering analysis of spatial transcriptomics data

**DOI:** 10.1093/bioadv/vbad019

**Published:** 2023-02-17

**Authors:** Xiao Zhang, Wei Liu, Fangda Song, Jin Liu

**Affiliations:** Centre for Quantitative Medicine Health Services & Systems Research, Duke-NUS Medical School, 169857 Singapore, Singapore; Centre for Quantitative Medicine Health Services & Systems Research, Duke-NUS Medical School, 169857 Singapore, Singapore; School of Data Science, The Chinese University of Hong Kong-Shenzhen, Shenzhen 518172, Guangdong, China; Centre for Quantitative Medicine Health Services & Systems Research, Duke-NUS Medical School, 169857 Singapore, Singapore; School of Data Science, The Chinese University of Hong Kong-Shenzhen, Shenzhen 518172, Guangdong, China

## Abstract

**Summary:**

Emerging spatially resolved transcriptomics (SRT) technologies are powerful in measuring gene expression profiles while retaining tissue spatial localization information and typically provide data from multiple tissue sections. We have previously developed the tool SC.MEB—an empirical Bayes approach for SRT data analysis using a hidden Markov random field. Here, we introduce an extension to SC.MEB, denoted as integrated spatial clustering with hidden Markov random field using empirical Bayes (iSC.MEB) that permits the users to simultaneously estimate the batch effect and perform spatial clustering for low-dimensional representations of multiple SRT datasets. We demonstrate that iSC.MEB can provide accurate cell/domain detection results using two SRT datasets.

**Availability and implementation:**

iSC.MEB is implemented in an open-source R package, and source code is freely available at https://github.com/XiaoZhangryy/iSC.MEB. Documentation and vignettes are provided on our package website (https://xiaozhangryy.github.io/iSC.MEB/index.html).

**Supplementary information:**

Supplementary data are available at *Bioinformatics Advances* online.

## 1 Introduction

Recently developed spatially resolved transcriptomics (SRT) technologies enable researchers to simultaneously characterize the gene expression profiles and their physical locations with a growing spatial resolution, providing extraordinary opportunities for researchers to investigate the spatial landscape of transcriptomic profiles within solid tissues. Among various analyses and applications using SRT datasets, identifying the cell/domain clusters is an essential analytic step. Several methods have been developed to make use of spatial information to enhance cell/domain type clustering, including a fully Bayesian statistical method, BayesSpace (Zhao *et al.*, 2021), a graph convolutional network approach, SpaGCN (Hu *et al.*, 2021), and an empirical Bayes method using hidden Markov random fields, SC-MEB (Yang *et al.*, 2021). Those methods were primarily designed to perform spatial clustering for SRT datasets from a single slide. When SRT datasets from multiple slides are available, it is desired that different spatial clustering methods are able to remove unwanted variations from batches. Although a recently developed method, BASS (Li and Zhou, 2022), enables multi-sample analysis for SRT data using embeddings aligned from Harmony (Korsunsky *et al.*, 2019), Harmony itself performs data integration in embeddings without any consideration of spatial information.

To perform the clustering analysis for multiple SRT datasets, we develop iSC.MEB, a flexible, computationally inexpensive, multi-threaded and user-friendly R package that supports multiple SRT datasets integration clustering in a unified step. iSC.MEB was implemented via an efficient expectation (E)–maximization (M) algorithm based on an iterative conditional mode, and selected the number of clusters by modified Bayesian information criterion (Wang *et al.*, 2009). The flexibility of iSC.MEB in modeling spatial dependency is achieved by accounting for local microenvironments of neighboring spots through a CAR model (Besag, 1974) and adjusting the spatial smoothness adaptively. The cell/domain labels and the slide-specific embeddings provided by iSC.MEB can facilitate many downstream analyses.

## 2 Methods

### 2.1 Approach

**Fig. 1.**
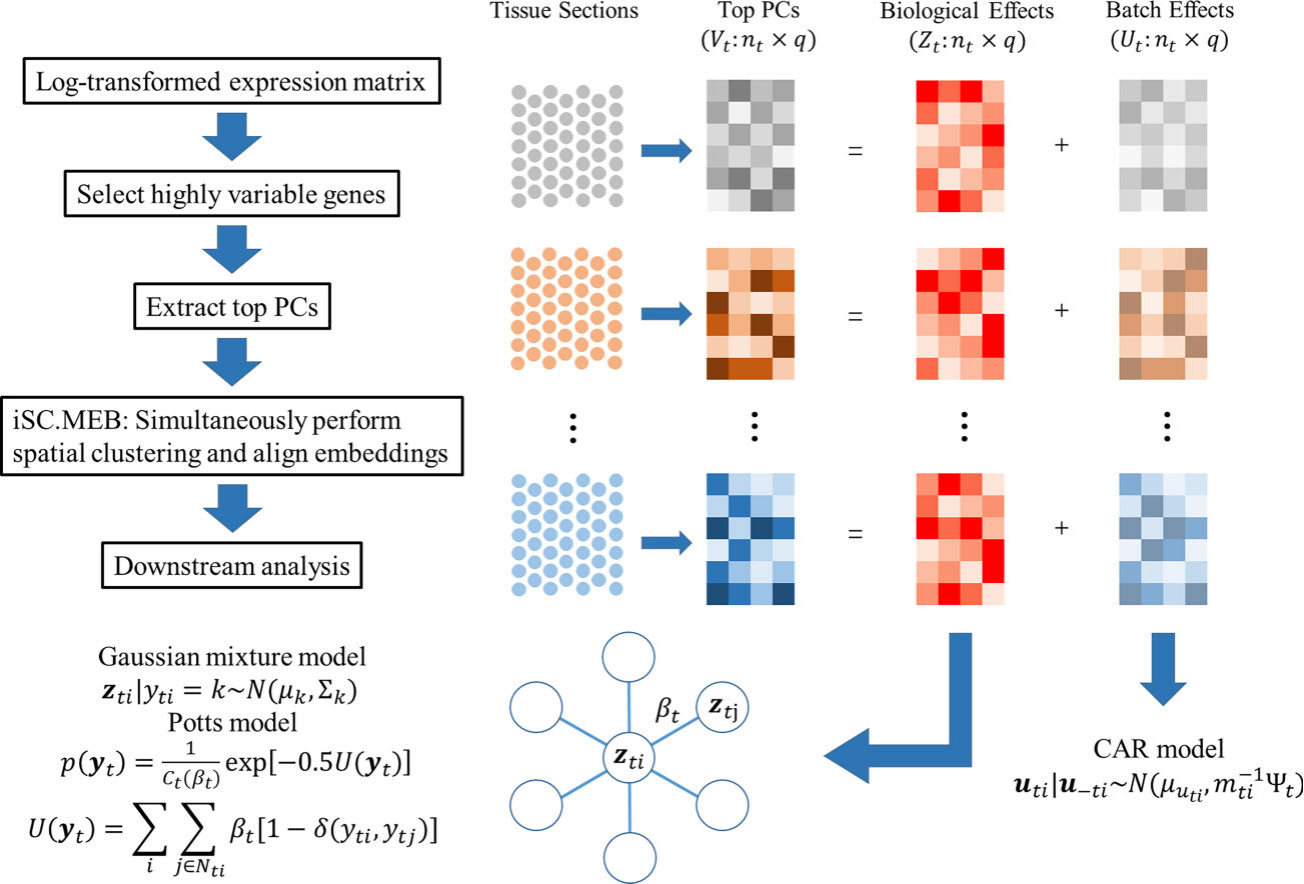
Schematic overview of iSC.MEB. iSC.MEB is a unified hidden MRF model that simultaneously estimates aligned embeddings and cluster labels with consideration of spatial smoothness in both the cluster label and batch effects

The iSC.MEB method is designed for integrative clustering analysis on the low-dimensional representations of multiple spatial transcriptomics datasets. A workflow outlining the general steps of the iSC.MEB is provided in Figure 1. First, we perform principal component analysis (PCA) on the combined log-transformed expression matrix to obtain the top principal components (PCs). Second, by taking the top PCs as input, we perform the integrative spatial clustering analysis using iSC-MEB that produces the soft cluster assignment as output. Finally, we can perform downstream analysis using the clustering result from the second step.

In iSC.MEB, for the spot *i* comes from tissue *t*, its low-dimensional representation $v_{ti}$ is considered to consist of two parts, the cluster-related embedding $z_{ti}$ and the batch-related embedding $u_{ti}$. We model the cluster-related part by a two-layer hierarchical probabilistic model. In brief, the first layer specific the conditional distribution of $z_{ti}$ given its unknown label $y_{ti}$. And in the second layer, a Potts model (Cuadra *et al.*, 2005) is adopted to promote spatial smoothness in the cluster label space. In addition, a conditional autoregressive (CAR) model (Besag, 1974) is imposed on the batch-related part to capture the spatial dependence induced by neighboring microenvironments. Please refer to Supplementary Section S1 for details.

To make the computation tractable, iSC.MEB updates parameter through an ICM-EM algorithm (Cuadra *et al.*, 2005). Assumes the number of clusters, *K*, is known. In the ICM step, we alternate between estimate label $y_{ti}$ by maximizing its posterior given $u_{ti}={\hat{u}}_{ti}$ and estimate the batch-related embedding $u_{ti}$ by maximizing its posterior given $y_{ti}={\hat{y}}_{ti}$. Then, in the E step, we derive the evidence lower bound (ELBO) with respect to the pseudo-observed log-likelihood. By taking partial derivatives of the ELBO, we obtain the updated equations in the M step. The smoothness parameter $\beta_{t}$ is updated via a grid search strategy since there is no closed-form solution for it. The ICM-EM algorithm iterates the ICM step and M step until convergence, resulting in parameter estimate and corresponding log-likelihood. Once the log-likelihoods for a sequence of K are obtained, MBIC is adopted to choose the optimal number of clusters. Further details on the ICM-EM algorithm and the MBIC are provided in Supplementary Section S2.

### 2.2 Implementation

iSC.MEB contains four main functions: ‘CreateISCMEBObject’, ‘runPCA’, ‘CreateNeighbors’ and ‘iSCMEB’. In detail, the user first uses ‘CreateISCMEBObject’ that takes as input a list of Seurat objects (Hao *et al.*, 2021) to perform the preprocessing steps and creates an object that contains all the data needed for subsequent modeling. By interfacing with common data formats and combining tedious preprocessing steps into a single function, our package facilitates integrative clustering analysis for users. ‘runPCA’ generates top PCs with three optional dimension reduction methods: the classical PCA, the approximate PCA (Baglama and Reichel, 2005) and the weighted PCA (Hong *et al.*, 2018). The default method is the approximate PCA to speed up the initial value calculation. ‘CreateNeighbors’ provides a platform-free way to generate an adjacency matrix by position information, which supports the application of our approach to emerging new SRT data. The model is trained in ‘iSCMEB’, with computational core written in C to improve computational efficiency. In addition, to make full use of multi-core computing resources, a parallel strategy is adopted to fit the model with a sequence of the number of clusters, *K*. Finally, our package also provides functions for downstream analysis, such as visualization of clusters and embeddings and combined differential expression analysis.

## 3 Results

We applied iSC.MEB to two real datasets and made comparisons with existing methods of clustering (Supplementary Section S3), including (i) BASS (Li and Zhou, 2022), (ii) SC.MEB (Yang *et al.*, 2021), (iii) BayesSpace (Zhao *et al.*, 2021), (iv) SpaGCN (Hu *et al.*, 2021) and (v) Louvain (Blondel *et al.*, 2008). Among these methods, BASS relies on Harmony to remove batch effects from multiple slides and applies spatial clustering on the aligned embeddings from Harmony, while other methods are used for clustering on a single slide. The manual annotations provided by original studies were taken as ground truth, which allowed us to evaluate the performance of both the accuracy of spatial domain detection and data integration.

**Fig. 2.**
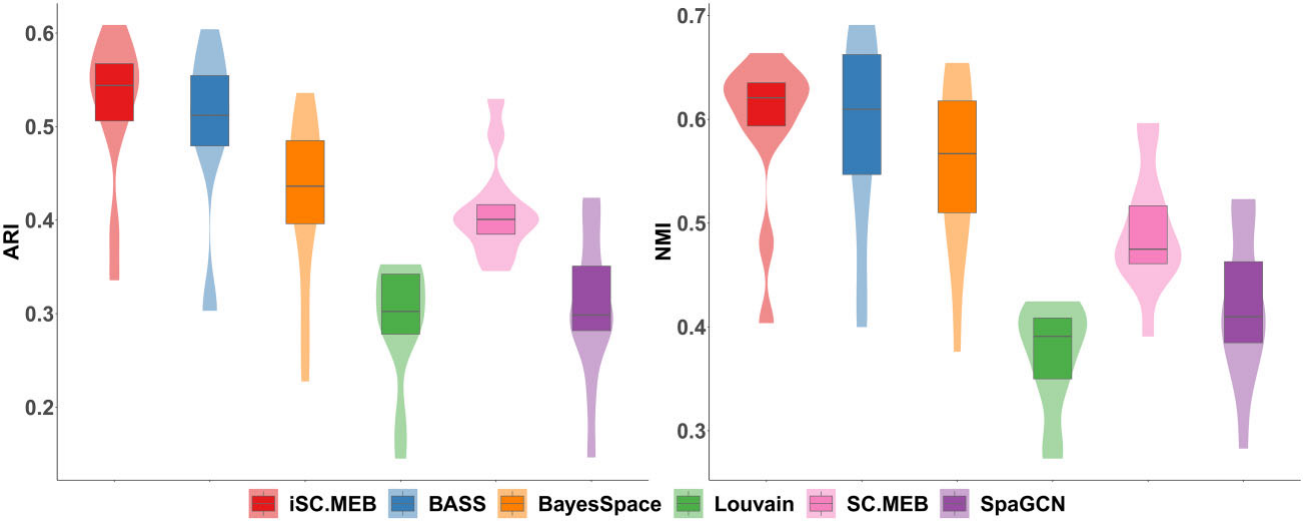
Summary of clustering accuracy for DLPFC dataset. ARI and NMI are evaluated by comparing manual annotations against cluster labels from iSC.MEB and benchmarks for each tissue section

The dorsolateral prefrontal cortex (DLPFC) dataset (Supplementary Section S3.1) contains the spatial topography of gene expression from 12 human postmortem DLPFC tissue sections generated by using the 10× Genomics Visium platform (Maynard *et al.*, 2021). The expression count matrix contained 47 681 spatial locations, with 33 538 genes for each spot. Figure 2 shows the adjusted rand index and normalized mutual information (Luecken *et al.*, 2022), abbreviated as ARI and NMI, respectively, for 12 DLPFC samples. iSC.MEB provided a higher median of ARI and NMI values than all other methods, indicating its superior clustering performance. And in general, multi-sample analysis methods outperformed single-slide analysis methods, demonstrating the benefits of multiple datasets integration to improve clustering accuracy. As shown in Supplementary Figure S1, the speed of iSC.MEB was significantly more efficient than that of BASS and BayesSpace, and comparable to SC.MEB. Both BASS and BayesSpace were implemented by MCMC algorithm, and their high computational cost may limit their application. Louvain and SpaGCN were faster, but their clustering accuracy was inferior. In addition, the integrated ARI and integrated NMI were (0.48,0.57) for iSC.MEB, showing that iSC.MEB achieves better data integration performance than BASS (see Supplementary Table S1).

**Fig. 3.**
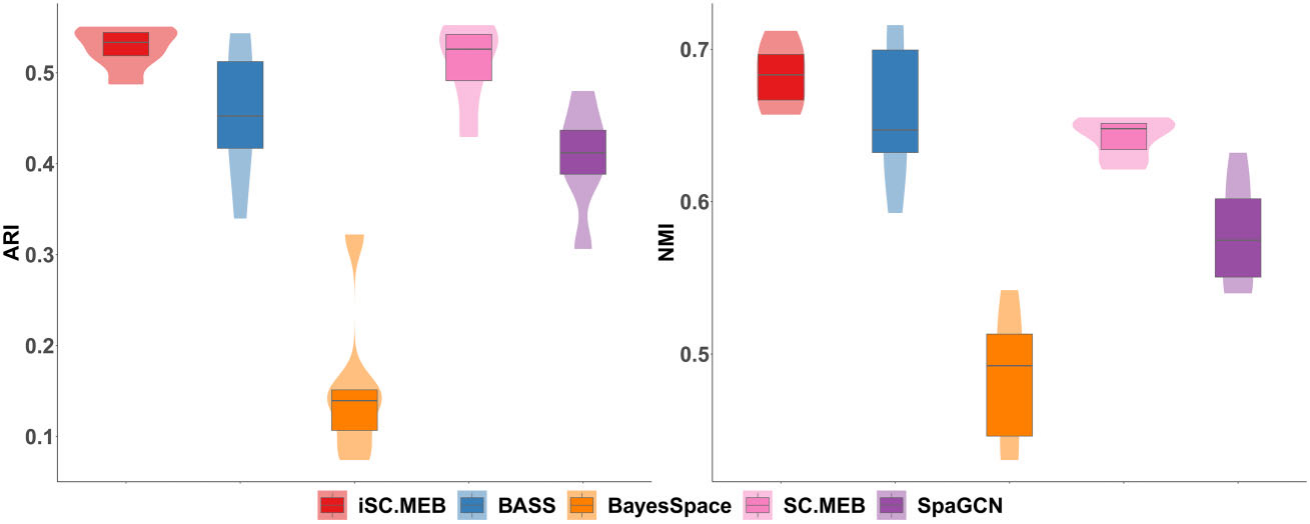
Summary of clustering accuracy for seqFISH dataset. ARI and NMI are evaluated by comparing provided annotations against cluster labels from iSC.MEB and benchmarks for each tissue section

The embryo dataset (Supplementary Section S3.2) contains the high-resolution spatial map from six mouse embryo tissue sections generated by a modified version of the seqFISH (sequential fluorescence *in situ* hybridization) method which allows highly effective cell segmentation (Lohoff *et al.*, 2021). The expression count matrix contained 387 selected target genes for each mRNA spot, and after cell-level quality control, we obtain a total of 51 800 spatial locations. Since the annotation procedure for these embryo datasets was based on Louvain network clustering, we did not compare its clustering performance using Louvain. The ARI values and NMI scores obtained using different methods are shown in Figure 3. Clearly, iSC.MEB provided the highest median of ARI and NMI values among all benchmarks. The integrated ARI and integrated NMI for iSC.MEB were (0.52,0.69), while those for BASS were (0.45,0.66). The runtime results demonstrated similar patterns to those obtained on DLPFC dataset (Supplementary Fig. S2).

## 4 Conclusion

We presented an extension to the existing method SC.MEB, denoted as iSC.MEB, which is an efficient R package for integrating low-dimension embeddings from multiple spatial transcriptomics datasets with batch effects. Compared with existing approaches on two real data analyses, iSC.MEB provides more accurate integration results. By applying iSC.MEB, the fitted cluster labels, and aligned embeddings can be applied for downstream analysis, such as trajectory analysis and visualization.

## Supplementary Material

vbad019_Supplementary_Data

## Data Availability

All datasets used in this study are publicly available. These include the twelve human dorsolateral prefrontal cortex Visium datasets (https://github.com/LieberInstitute/spatialLIBD) and six mouse embryo seqFISH datasets (https://content.cruk.cam.ac.uk/jmlab/SpatialMouseAtlas2020/).
