# Supplementary material for: iSC.MEB: an R package for multi-sample spatial clustering analysis of spatial transcriptomics data
Source: Bioinform Adv. 2023 Feb 17;3(1):vbad019. doi: 10.1093/bioadv/vbad019 (PMC9945056; doi:10.1093/bioadv/vbad019)
Supplement: vbad019_Supplementary_Data [file vbad019_supplementary_data.pdf]

iSC.MEB: an R package for multi-sample spatial clustering analysis of spatial transcriptomics data

Xiao Zhang[1], Wei Liu[1], Fangda Song[2], and Jin Liu[1,2]

[1] Centre for Quantitative Medicine, Health Services & Systems Research, Duke-NUS Medical School, 8 College Road, 169857, Singpore, Singpore
[2] School of Data Science, The Chinese University of Hong Kong-Shenzhen, 2001 Longxiang Avenue, 518172, Guangdong, China

February 9, 2023

1 Spatial clustering model

iSC.MEB is designed for modeling the low-dimensional representations of gene expression matrices from multiple tissue slides. Our model can be expressed as follows, for spot i in sample t, $t \in \{1, \cdots, T\}, i \in \{1, \cdots, n_r\}$, we have

$$\begin{aligned} \boldsymbol{v}_{ti} &= \boldsymbol{z}_{ti} + \boldsymbol{u}_{ti}, \\ \boldsymbol{z}_{ti}|y_{ti} &= k \sim \mathcal{N}(\boldsymbol{\mu}_k, \boldsymbol{\Sigma}_k), k \in \{1, \cdots, K\} \end{aligned} \tag{1.1}$$

where $\boldsymbol{v}_{ti} \in \mathcal{R}^q$ is the low-dimensional embedding, $\boldsymbol{z}_{ti} \in \mathcal{R}^q$ is the latent variable that captures cluster information, $\boldsymbol{u}_{ti} \in \mathcal{R}^q$ is the latent variable that captures batch effects, $y_{ti} \in \mathcal{R}$ is the latent class label, K is the pre-determined number of categories, $\boldsymbol{\mu}_k \in \mathcal{R}^q$ and $\boldsymbol{\Sigma}_k \in \mathcal{R}^{q \times q}$ are the mean and covariance matrix for the k-th cluster, respectively. To promote spatial smoothness in cluster label space, we assume that each latent class label, y_{ti}, is interconnected with the class labels of its neighborhoods through a discrete hidden Markov random field (HMRF). Briefly, the label vector of tissue t, $\boldsymbol{y}_t$, is assumed to follows

a Potts model,

$$\mathrm{P}(\boldsymbol{y}_t) = C_t(\beta_t)^{-1} \exp\left\{-\frac{1}{2}\sum_{i=1}^{n_t}\sum_{j\in N_{ti}} \beta_t(1-\delta(y_{ti}, y_{tj}))\right\}, \tag{1.2}$$

where δ is a Dirac function, $C_t(\beta_t)$ is a normalization constant, N_{ti} is the neighborhood of spot i in tissue t, and β_t is the smoothing parameter that controls the similarity among the neighboring labels, in other words, the degree of spatial smoothness. In addition, we assume a continuous multivariate HMRF for $\boldsymbol{u}_{ti}$ to captures the spatial dependence in the embedding space. In detail, $\boldsymbol{u}_{ti}$ is assumed to follows the CAR model (Besag 1974),

$$\boldsymbol{u}_{ti}|\boldsymbol{u}_{-ti} \sim \mathcal{N}(\boldsymbol{\mu}_{u_{ti}}, m_{ti}^{-1}\boldsymbol{\Psi}_t), \tag{1.3}$$

where subscript $_{-ti}$ denotes all spots except spot i in tissue t, m_{ti} is the number of neighborhoods of spot i in tissue t, $\boldsymbol{\mu}_{u_{ti}} = m_{ti}^{-1}\sum_{j\in N_{ti}} \boldsymbol{u}_{tj}$ and $\boldsymbol{\Psi}_t \in \mathcal{R}^{q\times q}$ is the covariance matrix of $\boldsymbol{u}_{ti}$.

Denote $\boldsymbol{\theta} = (\boldsymbol{\mu}_k, \boldsymbol{\Sigma}_k, k \in \{1,\cdots,K\}, \boldsymbol{\Psi}_t, \beta_t, t \in \{1,\cdots,T\})$ for all parameters in the model. Define $\boldsymbol{Y} = (\boldsymbol{y}_t, t \in \{1,\cdots,T\})$ as the collection of all labels. Let $\boldsymbol{v}_t = (\boldsymbol{v}_{t1},\cdots,\boldsymbol{v}_{tn_t})^{\mathsf{T}}$ be the low-dimensional embedding of sample t, $\boldsymbol{V} = (\boldsymbol{v}_t, t \in \{1,\cdots,T\})$ be the collection of all low-dimensional representations. $\boldsymbol{z}_t, \boldsymbol{Z}, \boldsymbol{u}_t$ and $\boldsymbol{U}$ are similarly defined. Combining (1.1), (1.2) and (1.3), we can obtain the log complete data likelihood,

$$\ln \mathrm{P}(\boldsymbol{V}, \boldsymbol{Z}, \boldsymbol{U}, \boldsymbol{Y}; \boldsymbol{\theta}) = \sum_t\sum_i [\ln \mathrm{P}(\boldsymbol{v}_{ti}|\boldsymbol{z}_{ti}, \boldsymbol{u}_{ti}) + \ln \mathrm{P}(\boldsymbol{z}_{ti}|\boldsymbol{y}_{ti}) + \ln \mathrm{P}(\boldsymbol{y}_{ti}) + \ln \mathrm{P}(\boldsymbol{u}_{ti})]. \tag{1.4}$$

To make the computation tractable, we define the pseudo-likelihood of $\boldsymbol{y}_t$ as

$$\tilde{\mathrm{P}}(\boldsymbol{y}_t) = \prod_{i=1}^{n_t} \mathrm{P}(y_{ti}|\hat{\boldsymbol{y}}_{-ti}), \tag{1.5}$$

and the pseudo-likelihood of $\boldsymbol{u}_{ti}$ as

$$\boldsymbol{u}_{ti}|\hat{\boldsymbol{u}}_{-ti} \sim \mathcal{N}(m_{ti}^{-1}\sum_{j\in N_{ti}} \hat{\boldsymbol{u}}_{tj}, m_{ti}^{-1}\boldsymbol{\Psi}_t). \tag{1.6}$$

By the approach, we simplify the spatial dependence structure and define the pseudo observed log-likelihood as

$$\begin{aligned}&\ln \tilde{\mathrm{P}}(\boldsymbol{V},\boldsymbol{Z},\boldsymbol{U},\boldsymbol{Y};\boldsymbol{\theta})\\=&\sum_t\sum_i[\ln \mathrm{P}(\boldsymbol{v}_{ti}|\boldsymbol{z}_{ti},\boldsymbol{u}_{ti})+\ln \mathrm{P}(\boldsymbol{z}_{ti}|\boldsymbol{y}_{ti})+\ln \mathrm{P}(y_{ti}|\hat{\boldsymbol{y}}_{-ti})+\ln \tilde{\mathrm{P}}(\boldsymbol{u}_{ti}|\hat{\boldsymbol{u}}_{-ti})].\end{aligned} \tag{1.7}$$

2 ICM-EM algorithm

To optimize the pseudo observed log-likelihood (1.7), we adopt an efficient iterative-conditional-mode-based expectation-maximization (ICM-EM) strategy (Cuadra et al. 2005) to update parameters.

In the ICM step, we first estimate y_{ti} by maximizing its posterior given $\boldsymbol{u}_{ti}=\hat{\boldsymbol{u}}_{ti}$. By Bayesian formula, we obtain

$$\mathrm{P}(y_{ti}|\hat{\boldsymbol{u}}_{ti},\hat{y}_{-ti},\boldsymbol{v}_{ti})\propto \mathrm{P}(\boldsymbol{v}_{ti}|\hat{\boldsymbol{u}}_{ti},y_{ti})\mathrm{P}(y_{ti}|\hat{y}_{-ti}). \tag{2.1}$$

Therefore, we can update y_{ti} through

$$\hat{y}_{ti}=\operatorname*{arg\,max}_{y_{ti}=1,\cdots,K}\mathrm{P}(\boldsymbol{v}_{ti}|\hat{\boldsymbol{u}}_{ti},y_{ti})\mathrm{P}(y_{ti}|\hat{y}_{-ti}). \tag{2.2}$$

Then, we estimate $\boldsymbol{u}_{ti}$ by maximizing its posterior given $y_{ti}=\hat{y}_{ti}$. The Bayesian formula shows that

$$\mathrm{P}(\boldsymbol{u}_{ti}|\hat{y}_{ti},\hat{\boldsymbol{u}}_{-ti},\boldsymbol{v}_{ti})\propto \mathrm{P}(\boldsymbol{v}_{ti}|\boldsymbol{u}_{ti},\hat{y}_{ti})\mathrm{P}(\boldsymbol{u}_{ti}|\hat{\boldsymbol{u}}_{-ti}). \tag{2.3}$$

Thus, the updated equation for $\boldsymbol{u}_{ti}$ is

$$\hat{\boldsymbol{u}}_{ti}=\operatorname*{arg\,max}_{\boldsymbol{u}_{ti}}\mathrm{P}(\boldsymbol{v}_{ti}|\boldsymbol{u}_{ti},\hat{y}_{ti})\mathrm{P}(\boldsymbol{u}_{ti}|\hat{\boldsymbol{u}}_{-ti})=F(\hat{y}_{ti}), \tag{2.4}$$

where

$$F(k)=\left(\boldsymbol{\Sigma}_k^{-1}+\boldsymbol{\Psi}_t^{-1}\right)^{-1}\left(\boldsymbol{\Sigma}_k^{-1}(\boldsymbol{v}_{ti}-\boldsymbol{\mu}_k)+\boldsymbol{\Psi}_t^{-1}\boldsymbol{\mu}_{u_{ti}}\right).$$

Finally, we repeat equation (2.2) and (2.4) until converge.

In the expectation (E) step, by the pseudo observed log-likelihood (1.7), we can derive its evidence lower bound (ELBO) is

$$Q(\boldsymbol{\theta}) = \sum_t \sum_i E_{\boldsymbol{\theta}^{(s)}} \left\{ \ln \frac{\mathrm{P}(\boldsymbol{v}_{ti}|\boldsymbol{z}_{ti}, \boldsymbol{u}_{ti})\mathrm{P}(\boldsymbol{z}_{ti}|\boldsymbol{y}_{ti})\mathrm{P}(y_{ti}|\hat{\boldsymbol{y}}_{-ti})\tilde{\mathrm{P}}(\boldsymbol{u}_{ti}|\hat{\boldsymbol{u}}_{-ti})}{\mathrm{P}(y_{ti}, \boldsymbol{z}_{ti}, \boldsymbol{u}_{ti}|\hat{\boldsymbol{y}}_{-ti}, \hat{\boldsymbol{u}}_{-ti}, \boldsymbol{v}_{ti}, \boldsymbol{\theta}^{(s)})} \right\}, \tag{2.5}$$

where expectation $E_{\boldsymbol{\theta}^{(s)}}$ is taken with respect to $(y_{ti}, \boldsymbol{z}_{ti}, \boldsymbol{u}_{ti})$ given $\hat{\boldsymbol{y}}_{-ti}, \hat{\boldsymbol{u}}_{-ti}, \boldsymbol{v}_{ti}$ and parameters $\boldsymbol{\theta}^{(s)}$. By Bayes' Formula, we have

$$\mathrm{P}(y_{ti}, \boldsymbol{z}_{ti}, \boldsymbol{u}_{ti}|\hat{\boldsymbol{y}}_{-ti}, \hat{\boldsymbol{u}}_{-ti}, \boldsymbol{v}_{ti}, \boldsymbol{\theta}^{(s)}) = \gamma_{tik}^{(s)} \mathrm{P}(\boldsymbol{z}_{ti}, \boldsymbol{u}_{ti}|\hat{\boldsymbol{y}}_{-ti}, \hat{\boldsymbol{u}}_{-ti}, \boldsymbol{v}_{ti}, \boldsymbol{\theta}^{(s)}, y_{ti} = k),$$

where

$$\gamma_{tik}^{(s)} = \frac{\mathrm{P}(\boldsymbol{v}_{ti}|y_{ti} = k, \hat{\boldsymbol{u}}_{ti}, \boldsymbol{\theta}^{(s)})\mathrm{P}(y_{ti} = k|\hat{\boldsymbol{y}}_t, \boldsymbol{\theta}^{(s)})}{\sum_{k'} \mathrm{P}(\boldsymbol{v}_{ti}|y_{ti} = k', \hat{\boldsymbol{u}}_{ti}, \boldsymbol{\theta}^{(s)})\mathrm{P}(y_{ti} = k'|\hat{\boldsymbol{y}}_t, \boldsymbol{\theta}^{(s)})}.$$

Since $(\boldsymbol{z}_{ti}, \boldsymbol{u}_{ti})$ given $\hat{\boldsymbol{y}}_{-ti}, \hat{\boldsymbol{u}}_{-ti}, \boldsymbol{v}_{ti}, \boldsymbol{\theta}^{(s)}$ is a multivariate normal distribution, using the formula about the conditional expectation and covariance of multivariate normal distribution, we have

$$\boldsymbol{z}_{ti}, \boldsymbol{u}_{ti}|\hat{\boldsymbol{y}}_{-ti}, \hat{\boldsymbol{u}}_{-ti}, \boldsymbol{v}_{ti}, \boldsymbol{\theta}^{(s)} \sim \mathcal{N}\left(\boldsymbol{\mu}_{tik}, \boldsymbol{\Sigma}_{tik}\right)$$

where

$$\begin{aligned}
\boldsymbol{\mu}_{tik} &= \begin{pmatrix} \boldsymbol{\mu}_{tik}^{z} \\ \boldsymbol{\mu}_{tik}^{u} \end{pmatrix} = \begin{pmatrix} \boldsymbol{\mu}_k \\ \hat{\boldsymbol{\mu}}_{u_{ti}} \end{pmatrix} + \begin{pmatrix} \boldsymbol{\Sigma}_k \\ \boldsymbol{\Psi}_t \end{pmatrix} \left(\boldsymbol{\Sigma}_k + \boldsymbol{\Psi}_t\right)^{-1} \left(\boldsymbol{v}_{ti} - \boldsymbol{\mu}_k - \hat{\boldsymbol{\mu}}_{u_{ti}}\right) \\
\boldsymbol{\Sigma}_{tik} &= \begin{pmatrix} \boldsymbol{\Sigma}_{tik}^{zz} & \boldsymbol{\Sigma}_{tik}^{zu} \\ \boldsymbol{\Sigma}_{tik}^{uz} & \boldsymbol{\Sigma}_{tik}^{uu} \end{pmatrix} = \begin{pmatrix} \boldsymbol{\Sigma}_k & \boldsymbol{0} \\ \boldsymbol{0} & \boldsymbol{\Psi}_t \end{pmatrix} - \begin{pmatrix} \boldsymbol{\Sigma}_k \\ \boldsymbol{\Psi}_t \end{pmatrix} \left(\boldsymbol{\Sigma}_k + \boldsymbol{\Psi}_t\right)^{-1} \begin{pmatrix} \boldsymbol{\Sigma}_k \\ \boldsymbol{\Psi}_t \end{pmatrix}^{\mathsf{T}}.
\end{aligned}$$

Taking derivatives of $Q(\boldsymbol{\theta})$ with respect to the parameter $\boldsymbol{\theta}$ and setting them to zero, we

obtain the updated equations in the maximization (M) step:

$$\boldsymbol{\mu}_k = \left\{ \sum_t \sum_i \gamma_{tik}^{(s)} \right\}^{-1} \left\{ \sum_t \sum_i \gamma_{tik}^{(s)} \boldsymbol{\mu}_{tik}^{z} \right\}, \tag{2.6}$$

$$\boldsymbol{\Sigma}_k = \left\{ \sum_t \sum_i \gamma_{tik}^{(s)} \right\}^{-1} \left\{ \sum_t \sum_i \gamma_{tik}^{(s)} \left[(\boldsymbol{\mu}_{tik}^{z} - \boldsymbol{\mu}_k)(\boldsymbol{\mu}_{tik}^{z} - \boldsymbol{\mu}_k)^{\mathsf{T}} + \boldsymbol{\Sigma}_{tik}^{zz} \right] \right\}, \tag{2.7}$$

$$\boldsymbol{\Psi}_t = n_t^{-1} \left\{ \sum_i \sum_k \gamma_{tik}^{(s)} \left[(\boldsymbol{\mu}_{tik}^{u} - \hat{\boldsymbol{\mu}}_{u_{ti}})(\boldsymbol{\mu}_{tik}^{u} - \hat{\boldsymbol{\mu}}_{u_{ti}})^{\mathsf{T}} + \boldsymbol{\Sigma}_{tik}^{uu} \right] \right\}. \tag{2.8}$$

Since there is no closed-form solution for β_t, we update the smoothness parameter β_t via a grid search strategy:

$$\beta_t = \underset{\beta_t \in \{b_1, \cdots, b_R\}}{\arg\max}\ Q(\boldsymbol{\theta}), \tag{2.9}$$

where the sequence $(b_1, \cdots, b_R)$ is a pre-determined search space of β_t.

The ICM-EM algorithm iterates the ICM step and M step until convergence.

2.1 MBIC criteria for determining K

In the implementation, iSC.MEB is implemented on a sequence of K, and then using MBIC (Wang et al. 2009) to determine the number of clusters K, which is given by

$$\text{MBIC}(K) = -2 \ln \text{P}(\boldsymbol{V}; \hat{\boldsymbol{\theta}}_K) + C_n \text{df}_K \ln n, \tag{2.10}$$

where df_K is the degree of freedom and C_n is a positive constant that can depend on n and q. Following Ma & Huang (2017), we let $C_n = c \ln(\ln(n + q))$, where c is a positive constant default as 1. Since the observed log-likelihood $\ln \text{P}(\boldsymbol{V}; \hat{\boldsymbol{\theta}}_K)$ is intractable, we use the pseudo observed log-likelihood (1.7) to approximate it.

3 Methods for comparison

We conducted real data analysis to compare SE-MEB2 with existing methods of data integration and clustering. The following methods are considered as benchmarks of perfor-

mance of iSC.MEB: (1) BASS (Li & Zhou 2022) implemented in the R package *BASS*; (2) SC.MEB (Yang et al. 2021) implemented in the R package *SC-MEB*; (3) BayesSpace (Zhao et al. 2020) implemented in the R package *BayesSpace*; (4) SpaGCN (Hu et al. 2021) implemented in the Python module *SpaGCN*; (5) Louvain (Blondel et al. 2008) implemented in the R package *igraph*. Among these methods, BASS is designed for scRNA-seq integration, while other methods are used for clustering on a single tissue.

In the analysis, we treated the manual annotations as the ground truth to evaluate the clustering and data integration performance of the different methods. In the implementation, iSC.MEB and SC.MEB are implemented on a sequence of K, and then using MBIC (Wang et al. 2009) to determine the optimal number of clusters in a data-driven manner. Louvain uses a community-modularity maximizing rule to choose the number of clusters. All other methods are implemented using the same number of clusters/domains as the manual annotations.

We evaluated the methods' performances by adjusted rand index (ARI, Hubert & Arabie (1985)) and normalized mutual information (NMI, Cover & Thomas (2006)). Both metrics are used for compares the overlap of two clusterings. ARI lies between -1 and 1, and a higher value means a higher degree of similarity between two partitions. NMI takes values from 0 to 1, and a NMI value of 1 indicate a perfect match. In addition, to evaluate the data integration clustering performance, we calculated ARI and NMI by comparing manual annotations against cluster labels on all samples, calling them integrated ARI (IARI) and integrated NMI (INMI), respectively.

3.1 Human dorsolateral prefrontal cortex Visium data

Recently, Maynard et al. (2021) generates the spatial topography of gene expression from 12 human postmortem DLPFC tissue sections by using the 10x Genomics Visium platform.

The expression count matrix contained 47,681 spatial locations, with 33,538 genes for each spot. The manual annotations of the layers based on the cytoarchitecture are also provided, which allowed us to evaluate the performance of both the data integration and accuracy of spatial domain detection by taking the manual annotations as ground truth. We first performed log-normalization of all datasets, and then obtain 15 top principal components (PCs) from 2,000 most highly variable genes. Finally, we performed clustering analysis for all methods.

Figure 1 shows the ARI values, NMI scores and running time for 12 DLPFC samples. iSC.MEB provided higher median of ARI and NMI values than all other methods, indicates its superior clustering performance. In addition, Figure 1 indicates that multi-sample analysis methods, iSC.MEB and BASS, gain an advantage from integration, and provide significantly better accuracy than single tissue section analysis methods. iSC.MEB, compared to BASS, had a higher ARI on 8 samples while having a comparable NMI. And in Figure 1, we can see that the computation time of iSC.MEB is significantly more efficient than BASS and BayesSpace. Louvain and SpaGCN were faster, but their clustering accuracy is inferior. Furthermore, as shown in Table 1, the IARI and INMI were $(0.48, 0.57)$ for iSC.MEB and $(0.43, 0.53)$ for BASS, which indicates that iSC.MEB has better data integration performance than BASS. Note that BASS was performed with the 'true' number of clusters from manual annotations, iSC.MEB achieves better performance without this prior information.

Table 1: Clustering accuracy for DLPFC dataset. IARI scores and INMI scores were evaluated by comparing manual annotations against cluster labels from iSC.MEB and BASS for combined samples.

metrics	iSC.MEB	BASS
IARI	**0.48**	0.43
INMI	**0.57**	0.53

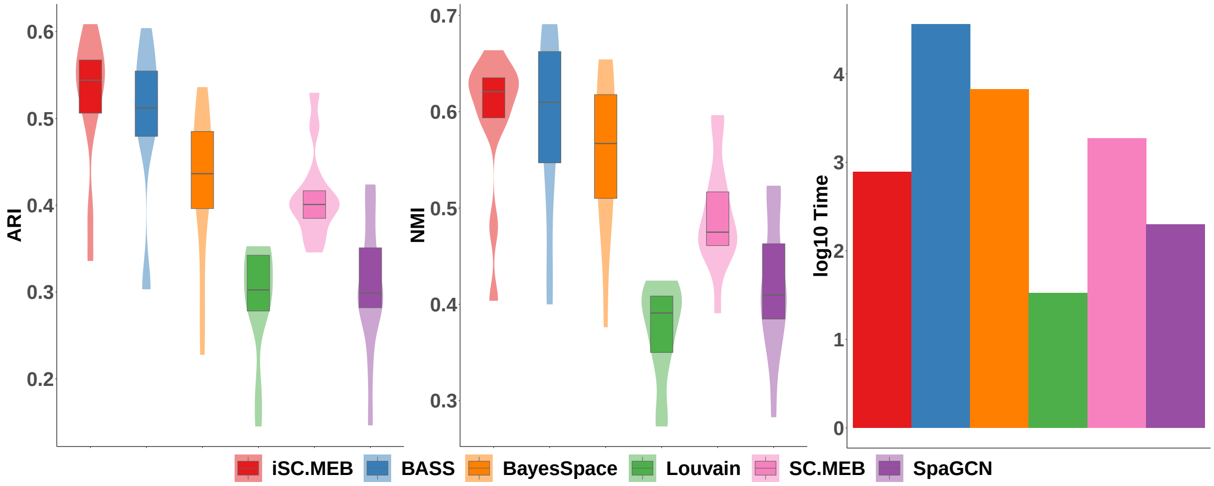

Figure 1: Summary of clustering accuracy and running time for DLPFC dataset. ARI and NMI are evaluated by comparing manual annotations against cluster labels from iSC.MEB and benchmarks for each tissue section.

3.2 Mouse embryo seqFISH data

Lohoff et al. (2020) provided the high-resolution spatial map from 6 mouse embryo tissue sections a modified version of the seqFISH (sequential fluorescence in situ hybridization) method which allow highly-effective cell segmentation. The expression count matrix contained 387 selected target genes for each mRNA spot, and after cell-level quality control, we obtain a total of 51,800 spatial locations. Its annotation procedure first perform joint analysis of seqFISH datasets and an existing scRNA-seq atlas (Pijuan-Sala et al. 2019) by constructing a shared nearest neighbor network on PCs followed by Louvain network clustering. Then, use the same method to find subclusters within each cluster. Finally, some subclusters are manual re-annotated based on the expression of marker genes and the relative contribution of cells to each subcluster. In the analysis, we treated these manual annotations provided by Lohoff et al. (2020) as the ground truth to evaluate the clustering and data integration performance of the different methods. We first obtain 15 top PCs using a similar approach to what we did for the DLPFC dataset. Then, the integrate spatial clustering performed by

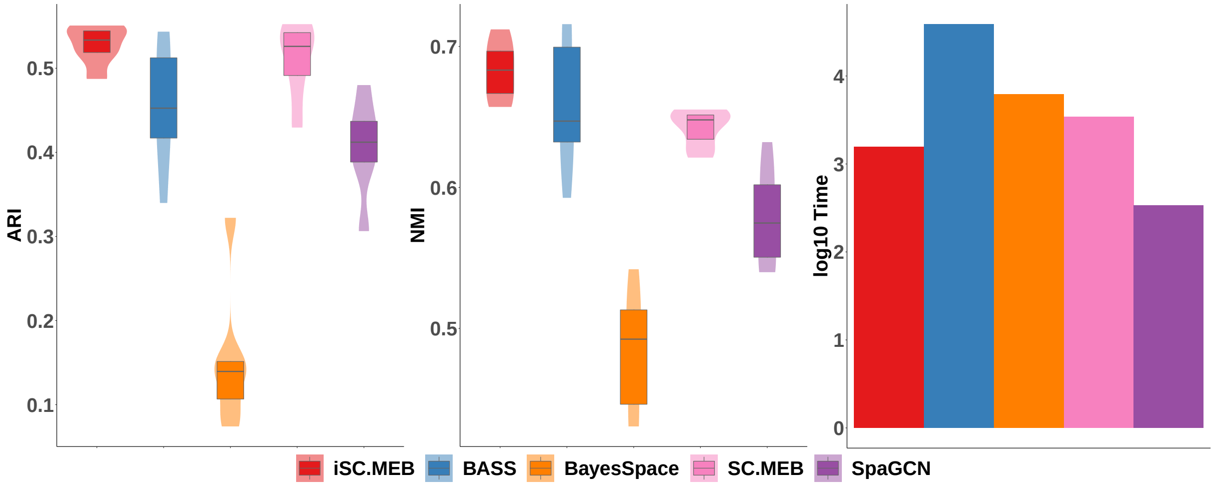

Figure 2: Summary of clustering accuracy and running time for seqFISH dataset. ARI and NMI are evaluated by comparing manual annotations against cluster labels from iSC.MEB and benchmarks for each tissue section.

iSC.MEB was compared with that of other methods except Louvain since Louvain was used for the annotation process of cell types.

The ARI values, NMI scores and running time obtained using different methods are shown in figure 2. iSC.MEB clearly outperform other methods in both ARI and NMI scores. The IARI and INMI for iSC.MEB were $(0.52, 0.69)$, while that for BASS were $(0.45, 0.66)$. The runtime results demonstrated similar patterns to those obtained on DLPFC dataset.

Table 2: Clustering accuracy for seqFISH dataset. IARI scores and INMI scores were evaluated by comparing manual annotations against cluster labels from iSC.MEB and BASS for combined samples.

metrics	iSC.MEB	BASS
IARI	**0.52**	0.45
INMI	**0.69**	0.66

References

Besag, J. (1974), 'Spatial interaction and the statistical analysis of lattice systems', *Journal of the Royal Statistical Society: Series B (Methodological)* **36**(2), 192–225.
URL: *https://doi.org/10.1111/j.2517-6161.1974.tb00999.x*

Blondel, V. D., Guillaume, J.-L., Lambiotte, R. & Lefebvre, E. (2008), 'Fast unfolding of communities in large networks', *Journal of Statistical Mechanics: Theory and Experiment* **2008**(10), P10008.
URL: *https://doi.org/10.1088/1742-5468/2008/10/p10008*

Cover, T. M. & Thomas, J. A. (2006), 'Elements of information theory 2nd edition (wiley series in telecommunications and signal processing)', *Acessado em* .

Cuadra, M., Cammoun, L., Butz, T., Cuisenaire, O. & Thiran, J.-P. (2005), 'Comparison and validation of tissue modelization and statistical classification methods in t1-weighted MR brain images', *IEEE Transactions on Medical Imaging* **24**(12), 1548–1565.
URL: *https://doi.org/10.1109/tmi.2005.857652*

Hu, J., Li, X., Coleman, K., Schroeder, A., Ma, N., Irwin, D. J., Lee, E. B., Shinohara, R. T. & Li, M. (2021), 'SpaGCN: Integrating gene expression, spatial location and histology to identify spatial domains and spatially variable genes by graph convolutional network', *Nature Methods* **18**(11), 1342–1351.
URL: *https://doi.org/10.1038/s41592-021-01255-8*

Hubert, L. & Arabie, P. (1985), 'Comparing partitions', *Journal of Classification* **2**(1), 193–218.
URL: *https://doi.org/10.1007/bf01908075*

Li, Z. & Zhou, X. (2022), 'BASS: multi-scale and multi-sample analysis enables accurate cell type clustering and spatial domain detection in spatial transcriptomic studies', *Genome Biology* **23**(1).
URL: *https://doi.org/10.1186/s13059-022-02734-7*

Lohoff, T., Ghazanfar, S., Missarova, A., Koulena, N., Pierson, N., Griffiths, J., Bardot, E., Eng, C.-H., Tyser, R., Argelaguet, R., Guibentif, C., Srinivas, S., Briscoe, J., Simons, B., Hadjantonakis, A.-K., Göttgens, B., Reik, W., Nichols, J., Cai, L. & Marioni, J. (2020), 'Highly multiplexed spatially resolved gene expression profiling of mouse organogenesis'.
URL: *https://doi.org/10.1101/2020.11.20.391896*

Ma, S. & Huang, J. (2017), 'A concave pairwise fusion approach to subgroup analysis', *Journal of the American Statistical Association* **112**(517), 410–423.

Maynard, K. R., Collado-Torres, L., Weber, L. M., Uytingco, C., Barry, B. K., Williams, S. R., Catallini, J. L., Tran, M. N., Besich, Z., Tippani, M., Chew, J., Yin, Y., Kleinman, J. E., Hyde, T. M., Rao, N., Hicks, S. C., Martinowich, K. & Jaffe, A. E. (2021), 'Transcriptome-scale spatial gene expression in the human dorsolateral prefrontal cortex', *Nature Neuroscience* **24**(3), 425–436.
URL: *https://doi.org/10.1038/s41593-020-00787-0*

Pijuan-Sala, B., Griffiths, J. A., Guibentif, C., Hiscock, T. W., Jawaid, W., Calero-Nieto, F. J., Mulas, C., Ibarra-Soria, X., Tyser, R. C. V., Ho, D. L. L., Reik, W., Srinivas, S., Simons, B. D., Nichols, J., Marioni, J. C. & Göttgens, B. (2019), 'A single-cell molecular map of mouse gastrulation and early organogenesis', *Nature* **566**(7745), 490–495.
URL: *https://doi.org/10.1038/s41586-019-0933-9*

Wang, H., Li, B. & Leng, C. (2009), 'Shrinkage tuning parameter selection with a diverg-

ing number of parameters', *Journal of the Royal Statistical Society: Series B (Statistical Methodology)* **71**(3), 671–683.
URL: *https://doi.org/10.1111/j.1467-9868.2008.00693.x*

Yang, Y., Shi, X., Liu, W., Zhou, Q., Lau, M. C., Lim, J. C. T., Sun, L., Ng, C. C. Y., Yeong, J. & Liu, J. (2021), 'SC-MEB: spatial clustering with hidden markov random field using empirical bayes', *Briefings in Bioinformatics* **23**(1).
URL: *https://doi.org/10.1093/bib/bbab466*

Zhao, E., Stone, M. R., Ren, X., Pulliam, T., Nghiem, P., Bielas, J. H. & Gottardo, R. (2020), 'BayesSpace enables the robust characterization of spatial gene expression architecture in tissue sections at increased resolution'.
URL: *https://doi.org/10.1101/2020.09.04.283812*
